# Phase changes in neuronal postsynaptic spiking due to short term plasticity

**DOI:** 10.1371/journal.pcbi.1005634

**Published:** 2017-09-22

**Authors:** Mark D. McDonnell, Bruce P. Graham

**Affiliations:** 1 Computational Learning Systems Laboratory, School of Information Technology and Mathematical Sciences, University of South Australia, Mawson Lakes, Australia; 2 Computing Science & Mathematics, Faculty of Natural Sciences, University of Stirling, Stirling, United Kingdom; University College London, UNITED KINGDOM

## Abstract

In the brain, the postsynaptic response of a neuron to time-varying inputs is determined by the interaction of presynaptic spike times with the short-term dynamics of each synapse. For a neuron driven by stochastic synapses, synaptic depression results in a quite different postsynaptic response to a large population input depending on how correlated in time the spikes across individual synapses are. Here we show using both simulations and mathematical analysis that not only the rate but the phase of the postsynaptic response to a rhythmic population input varies as a function of synaptic dynamics and synaptic configuration. Resultant phase leads may compensate for transmission delays and be predictive of rhythmic changes. This could be particularly important for sensory processing and motor rhythm generation in the nervous system.

## Author summary

The synapses that connect neurons in the brain are far from being simple relay points that pass a signal from one neuron to another. There is now much evidence that long term changes in the strength of such connections, which determines the amplitude of the received signal, underpin learning and memory in the brain. However, signal amplitudes also fluctuate on fast time scales of milliseconds to seconds due to a variety of particular presynaptic mechanisms that regulate the release of neurotransmitter from the presynaptic terminal. Understanding the signal filtering properties of this short-term plasticity (STP) is a challenge and requires theoretical models. Aspects such as rate filtering and information transfer have been studied. Here we explore the effects of STP on the phase of a receiving neuron’s response to oscillating input and show that short-term depression can result in a frequency-dependent phase lead. This may be particularly important in the processing of rhythmic visual and auditory signals and producing rhythmic motor outputs.

## Introduction

Chemical synapses between neurons in the brain exhibit short-term plasticity (STP) such that the postsynaptic signal produced by the arrival of an action potential at a presynaptic terminal is not identical for each action potential, but varies stochastically and functionally with respect to the presynaptic interspike interval [1–3]. Functional effects are predominantly either a depression of the response to successive spikes or a facilitation of the response. While such effects are an inevitable consequence of the complex signalling process at chemical synapses, it is also clear that the nervous system shapes STP according to postsynaptic target cells to produce suitable signal filtering [4]. Understanding the functional consequences of STP is a challenge which requires mathematical models to help solve. To this end, important advances have been made in elucidating the role that STP can play in cortical gain control, rate filtering and information transmission.

STP interacts with the presynaptic spike arrival times to produce an integrated postsynaptic response that is a dynamic function (temporal filter) of the incoming spike times. Over a history of one or a few presynaptic spikes, the excitatory postsynaptic current amplitude carries significant information (in the Shannon sense) about the presynaptic interspike intervals [5, 6]. This can be carried through to the spiking output of the receiving neuron [7, 8]. Synaptic depression with stochastic vesicle recycling results in a high-pass filter for information transfer [9]. Combinations of depressing and facilitating mechanisms with time constants ranging from milliseconds to seconds enable information transmission over a very wide range of interspike intervals [10].

On longer time scales (100 ms or more), over which the average firing rate of the inputs is relevant to driving output spiking, it has been demonstrated that synaptic depression leads to loss of rate information [11, 12]. This occurs because individual EPSPs decrease in amplitude as a function of the increase in presynaptic firing rate, with the consequence that the integrated postsynaptic response tends to a consistent amplitude over a range of input firing rates, and the output neuron fires at a constant rate regardless of the inputs. However, it has been demonstrated both with models and experimentally that this normalisation in firing rate can be exploited for gain control of neural responses [11, 13–15]. Changes in input firing rate (on the order of Hertz) cause transient increases or decreases in output firing rate as the processes of depression and recovery have time constants of the order of tens of milliseconds. Consequently, synaptic depression can allow a neuron to respond similarly to rhythmic changes in firing, independently of the mean input rate [11]. Other studies have shown that the output response amplitude is a function of input modulation frequency because of STP [9, 16, 17]. In addition, correlations in vesicle release, which may arise when multiple release sites are driven by the same presynaptic spike, can, up to a point, overcome synaptic saturation to improve rate transmission through depressing synapses [18, 19]. Synaptic facilitation can enhance the detection of firing rate changes and correlations between input streams [20]. A balance between facilitation and depression can lead to multiple stochastic resonance peaks for detecting a weak input signal imposed on random background firing at different rates through dynamic synapses [21, 22].

Models have been used to explore the effects of STP on neural network behaviour. STP can contribute to oscillatory activity and synchronous firing in networks [23, 24]. However, short-term depression with stochastic vesicle dynamics can act to reduce correlations between the output spike trains of neurons being driven by correlated inputs [25]. Cellular responses to population bursts can vary markedly depending on the depressing or facilitating nature of the synaptic connections [16, 26]. At a putative cognitive level, STP can strongly influence pattern recall behaviour in associative memory attractor neural networks. Synaptic facilitation can provide a “memory” of the previously recalled pattern, even in the absence of spiking activity [27]. During active recall, STP and stochastic vesicle dynamics can promote sensitivity to dynamic input patterns and cause switching between recall states [22, 28, 29]. Facilitation can restore memory capacity lost by the presence of synaptic depression [30].

Rhythms in the nervous system are often on time scales that match processes of depression and facilitation, so that the output response is almost purely determined by the STP transients, rather than instantaneous or steady state responses. Rhythmic motor behaviour is on the order of one to a few Hertz, and so is likely to be influenced in this way by STP. In oscillating networks, a combination of facilitation and depression can influence both the period of oscillations and the phases of the component neurons [31, 32]. Response phase can be particularly important in motor systems: in the vestibular-ocular reflex (VOR), eye movements need to counteract head rotations with minimal phase offset despite time delays through multiple synapses; swimming in the lamprey (and similar creatures) requires maintaining constant phase offsets between motor units along the body, despite changes in swimming speed [33]. Sensory stimuli can also be rhythmic, or at least transient. Modelling has been used to show that short-term depression can contribute to phase leads seen in visual system responses to temporally oscillating gratings [34]. Such phasic responses could contribute to the direction selectivity of visual cortex cells [34].

**Fig 1:**
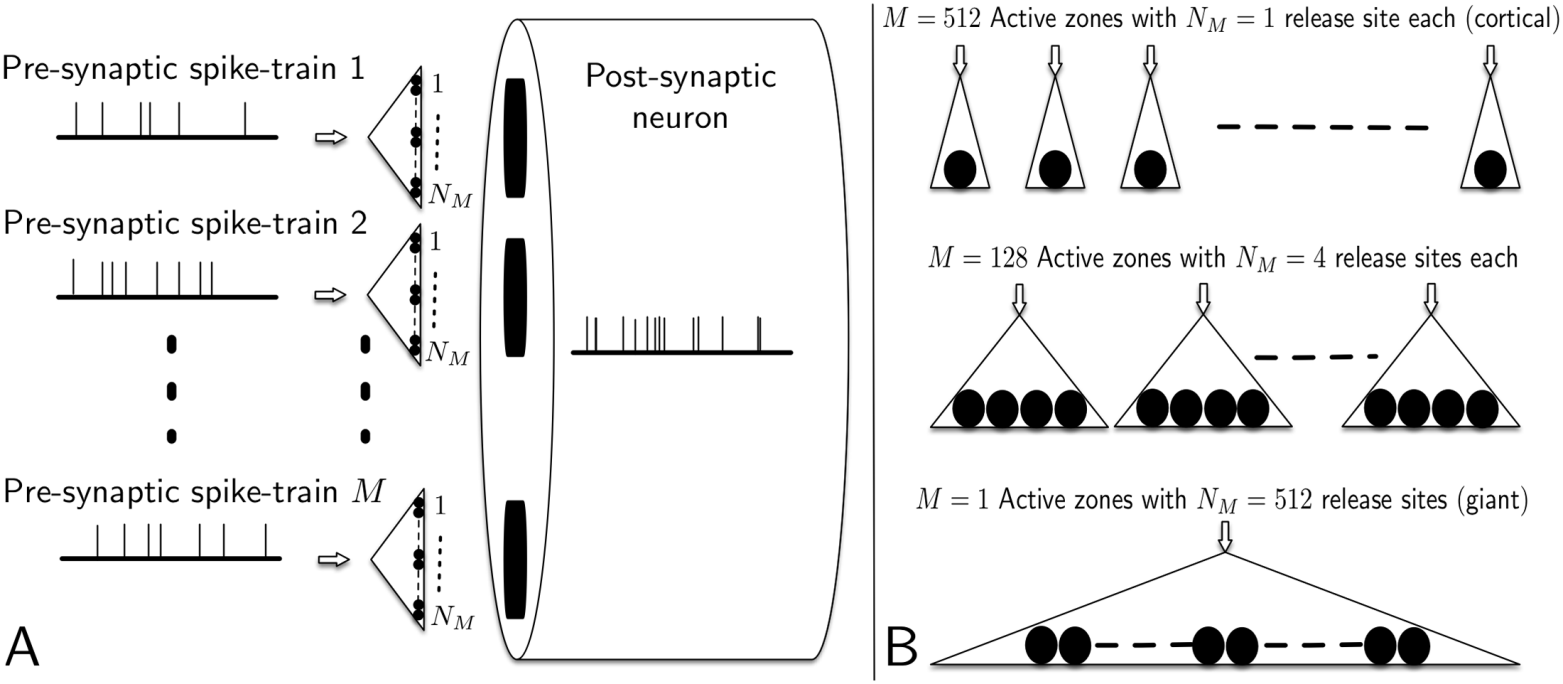
Conceptual diagram of the overall model and examples of different synaptic configurations. As shown in Subfigure A, we assume a total of *M* independent pre-synaptic neurons each make multiple synaptic contacts with a post-synaptic neuron. We refer to each pre-synaptic neuron as forming a single active-zone, and assume each active zone has *N*_*M*_ sites that each probabilistically release a single-vesicle when activated by a pre-synaptic action-potential, if one is available at that site. The post-synaptic neuron is assumed to be depolarized by arrival of neurotransmitter resulting from release of vesicles, according to standard models of AMPA receptors. Subfigure B shows a total of *N* = 512 vesicle release sites are equally distributed between *M* active zones. Each active zone is the target of a single presynaptic axon (arrows) being driven by a unique input neuron. We refer to the case of a single active zone (*M* = 1) as the “giant” synaptic pathway, and the case of 512 active zones (*M* = 512) as the “cortical” synaptic pathway.

In this paper we study the effect of STP on the phase of a neuron’s spiking response to rhythmic variations in the input firing rates, as might be found in motor and sensory systems, in particular. The aim is to clarify the specific contribution of synaptic depression to phasic responses as seen in other modelling studies [31, 34]. We examine in detail, using both mathematical analysis and numerical simulations, the relationship between input and output phases as a function of STP, during rhythmic stimulation. We consider the following two aspects of STP:

- We examine how STP affects the phase of the spiking output response of neurons driven by a frequency-modulated input signal and depressing synapses. The input signal is encoded as the firing rates of input neurons forming synapses with an output neuron (see Fig 1).
- We consider also the effect of the configuration of the synaptic pathway onto an output neuron. This pathway is assumed to consist of a fixed number of release sites that are divided between active zones, with each active zone being the presynaptic axonal target of a single input neuron (see Fig 1). For the same number of release sites, at one extreme a configuration consists of the output neuron receiving input from a large number of neurons through independent active zones, each containing a single release site. At the other extreme, the output neuron is driven by a single input neuron through a giant synapse containing a single active zone with a very large number of release sites. Both extremes, and variations in between, are present in the mammalian nervous system.

We conclude from these investigations that differences in synaptic configuration strongly determines the impact of STP on the phase of output neuron response. Moreover, we show that phase changes also depend strongly on the frequency of pre-synaptic modulation, but otherwise remains largely invariant for a wide range of post-synaptic conditions.

The paper is organised as follows. In Models and Methods, we describe the mathematical models we use to describe the input stimuli, the synaptic dynamics, and the post-synaptic neuron’s membrane potential, as well as outlining the methods used to simulate the model and measure spiking in the simulated post-synaptic neuron. Then, in Results we present and outline the results of our numerical experiments as various parameters in the model are altered. We also present supporting theoretical analysis of the underlying mathematical model. Finally, in Discussion, we discuss the implications for neurobiological function, and comment on possible extensions to our model.

## Models and methods

We model the feed-forward activation of a post-synaptic neuron due to pre-synaptic spiking in *M* pre-synaptic neurons. There are four components in our model:

1. modulated pre-synaptic spiking in *M* independent parallel neurons;
2. the synaptic connectivity between these *M* pre-synaptic neurons and a single post-synaptic neuron (see Fig 1);
3. vesicle release following arrival of spikes at each presynaptic release site;
4. changes in the post-synaptic neuron’s membrane potential due to neurotransmitter released by the pre-synaptic neurons.

We next describe the models we use for each of these components, conceptually and mathematically.

### Input stimulus

We model the input signal to all *M* pre-synaptic neurons as independent inhomogeneous (time-dependent) Poisson spike trains, each with a mean rate of 30 Hz. This mean rate is modulated sinusoidally to vary between instantaneous rates of 10 and 50 Hz, at different modulation frequencies, *f*, in the interval *f* ∈ [0.1, 5] Hz. We write the time-dependent spike-rate as

$$\begin{array}{c} \lambda_{S}\left(t\right)=A+B\sin\left(2\pi ft\right), \end{array}$$ (1)

where *A* = 30 Hz is the mean input spike frequency, and *B* = 20 Hz is the peak modulation of the input spike frequency.

This stimulus protocol mimics, for example, vestibular input to vestibular nucleus neurons during head rotations in the vestibulo-occular reflex (VOR), and the sinusoidal form makes possible the determination and analysis of the phase of the response. However, the results to be described below are relevant for any neural system in which changes in input rate occur on timescales of tens of milliseconds to seconds.

### Synaptic configuration

An axon from a presynaptic cell is assumed to make contact with the postsynaptic cell via a single active zone (AZ) that contains a specified number of release sites, *N*_*M*_. A synaptic pathway consists of a set of AZs, each driven by one of *M* different presynaptic cells. Different synaptic configurations are constructed from a fixed total number of release sites, *N*. Throughout this paper we assume *N* = 512. The release sites are assumed to be equally subdivided between a specified number of AZs (see Fig 1) so that *N*_*M*_ = *N*/*M*. Thus, configurations range from a single AZ (*M* = 1) containing *N*_*M*_ = 512 release sites, of the form of giant synapses such as the calyx of Held [36], to *M* = 512 AZs, each containing a single release site (*N*_*M*_ = 1), of the form of excitatory synaptic input onto cortical pyramidal cells from a population of presynaptic cells whose activity encodes a common stimulus [11]. We refer to these two extreme configurations as the “giant” and “cortical” synaptic pathways, respectively.

### Stochastic vesicle release and replenishment

We consider a simple standard model of vesicle availability, release and replenishment at individual presynaptic release sites [12, 37–39]. The available pool of readily-releasable vesicles (RRVP) is depleted by stochastic vesicle release on arrival of a presynaptic action potential. For each release site, we assume that only a single vesicle may be released at the time of each pre-synaptic spike.The RRVP recovers by replenishment from an infinitely large reserve pool. Hence, we assume that a single release site may either be occupied by a single releasable vesicle, or it may be empty. If a site in the RRVP is occupied, then the arrival of a pre-synaptic spike will cause that vesicle to be released with a constant probability *P*_v_. Following a vesicle release, we assume the time before the site is refilled with a vesicle from the reserve pool is random, with an exponential distribution, with replenishment time constant *τ*_rec_, and that the refill of each site is independent.

### Postsynaptic cell

To eliminate variability due to dendritic integration, we model all sites postsynaptic to an active zone as lying on the soma of the receiving cell. Thus the postsynaptic cell is modelled as a single cylindrical compartment, with length and diameter of 20 *μm*. Each presynaptic active zone corresponds to a postsynaptic specialisation containing a population of fast, excitatory AMPA channels. Release of a vesicle generates an excitatory postsynaptic current (EPSC), according to the model stated below (see Eq (8)).

In order to rule out a role for the post-synaptic action-potential generation mechanism as a cause for our findings, we consider two alternative models for the membrane potential of the post-synaptic neuron:

1. a Hodgkin-Huxley (HH) model, where active ionic currents are responsible for generating action potentials; note that rather than the use the original equations for a squid axon [40], we instead assume a cortically-relevant model where the membrane generates fast sodium and delayed-rectifier potassium currents [41];
2. a leaky-integrate and fire (LIF) model, where we choose parameters to approximately match the firing rates obtained from the HH model.

Note that the choice of parameters in our HH model means it is in the Class-II excitability mode, which is not the case for the LIF model [42], and hence we have two quite different dynamical systems for the post-synaptic neuron.

We now describe these two models in detail, followed by the model of EPSC generation. The values for all parameters in the models are shown in Tables 1 and 2.

**Table 1:** Parameters for the HH and LIF neuron models, corresponding to a cylinder of diameter 20*μ*m and length 20*μ*m.

Parameter	Notation	Value	Units
Membrane capacitance per square cm	*C*_m_	1	*μ*F.cm^−2^
Leak conductance per square cm	*g*_l_	2 × 10^−4^	S.cm^−2^
Peak potassium conductance per square cm (HH)	${\bar{g}}_{K}$	0.030	S.cm^−2^
Peak sodium conductance per square cm (HH)	${\bar{g}}_{\text{Na}}$	0.025	S.cm^−2^
Leak reversal potential	*E*_l_	-66	mV
Potassium reversal potential (HH)	*E*_K_	-95	mV
Sodium reversal potentual (HH)	*E*_Na_	50	mV
Time constant for *m* subunits (HH)	*τ*_*m*_	0.05	ms
Time constant for *h* subunits (HH)	*τ*_*h*_	0.5	ms
Time constant for *n* subunits (HH)	*τ*_*n*_	2	ms
Spiking threshold (LIF)	*v*_thresh_	-51.5	mV
Spiking threshold (LIF)	*v*_reset_	-80.0	mV
Refractory time (LIF)	*t*_refrac_	1.8	ms
Membrane area	*a*	1.2566 × 10^−5^	cm^2^
Synaptic reversal potential	*E*_synapse_	0	mV
Synaptic rise time	*τ*_rise_	0 or 0.1	ms
Synaptic decay time	*τ*_d_	1	ms

The parameters that are relevant only to the HH model are indicated as HH. These parameter values mostly come from [41]. The parameters that are relevant only to the LIF model are indicated as LIF. These parameter values are chosen to give similar LIF spiking characteristics to the HH model. Where no indication is given, the parameters are relevant to both models. The synaptic rise time has two values listed as we considered both cases. Note that the membrane time constant is equal to *C*_m_/*g*_l_ = 5 ms.

**Table 2:** Values of peak synaptic current for each value of *M* considered.

*M*	1	2	4	8	16	32	64	128	256	512
*w*_*M*_	0.12	0.17	0.23	0.29	0.32	0.35	0.38	0.4	0.41	0.42

The value of ${\bar{g}}_{\text{synapse}}$ is equal to *w*_*M*_ multiplied by a scaling of ${\left(\tau_{\text{rise}}/\tau_{d}\right)}^{\left(\tau_{\text{rise}}/\left(\tau_{d}-\tau_{\text{rise}}\right)\right)}=1.2915\times 10^{-9}$ S for *τ*_*r*_ = 0.1 ms (and 1 for *τ*_*r*_ = 0 ms).

#### Postsynaptic membrane potential: HH model

Our conceptual model is of a neuron with spatial extent, but since we consider a single compartment, the mathematical model is equivalent to a point model of the current per square centimetre across the membrane. The membrane potential, *v*(*t*), in *mV* depends on three time-dependent variables: *m*(*t*), *n*(*t*) and *h*(*t*), which represent subunit activations in the voltage-gated potassium and sodium channels (*m*(*t*) is the activated fraction and *h*(*t*) the inactivated fraction of sodium channels; *n*(*t*) is the activated fraction of the potassium channels, which do not inactivate), as in the standard Hodgkin-Huxley model. Hence, *v*(*t*) is the solution of the following four coupled differential equations [41]:

$$\begin{array}{c} C_{m}\frac{dv(t)}{dt}=-g_{l}\left(v\left(t\right)-E_{l}\right)-{\bar{g}}_{K}n{\left(t\right)}^{2}\left(v\left(t\right)-E_{K}\right)-{\bar{g}}_{\text{Na}}m{\left(t\right)}^{2}h\left(t\right)\left(v\left(t\right)-E_{\text{Na}}\right)+\frac{I_{\text{synapse}}\left(t\right)}{a}, \end{array}$$ (2)

$$\frac{dm(t)}{dt}=\frac{m_{\text{ss}}-m\left(t\right)}{\tau_{m}},\;m_{\text{ss}}≔\frac{1}{1+\exp(\frac{-(v(t)+40)}{3})},$$ (3)

$$\frac{dh(t)}{dt}=\frac{h_{\text{ss}}-h\left(t\right)}{\tau_{h}},\;h_{\text{ss}}≔\frac{1}{1+\exp(\frac{v(t)+45}{3})},$$ (4)

$$\frac{dn(t)}{dt}=\frac{n_{\text{ss}}-n\left(t\right)}{\tau_{n}},\;n_{\text{ss}}≔\frac{1}{1+\exp(\frac{-(v(t)+40)}{3})},$$ (5)

where all parameter values are listed and defined in Table 1.

#### Postsynaptic membrane potential: LIF model

For the LIF model the membrane potential between action potentials is given by the solution of the following equation,

$$\begin{array}{c} C_{m}\frac{dv(t)}{dt}=-g_{l}\left(v\left(t\right)-E_{l}\right)+\frac{I_{\text{synapse}}\left(t\right)}{a}. \end{array}$$ (6)

Consistent with standard LIF models [43, 44], at the times at which *v*(*t*) goes above a threshold, *v*_thresh_, the membrane potential is held constant at a reset value, *v*_reset_ for a refractory time of *t*_refrac_.

#### Net postsynaptic current

In Eqs (2) and (6), the term *I*_synapse_(*t*) represents the total net synaptic current into the cell induced from vesicle release from presynaptic neurons in the model and is given by

$$\begin{array}{c} I_{\text{synapse}}\left(t\right)={\bar{g}}_{\text{synapse}}g\left(t\right)\left(v\left(t\right)-E_{\text{synapse}}\right), \end{array}$$ (7)

where the reversal potential is listed in Table 1.

To calculate the time-course of the synaptic conductance, we use the difference-of-exponentials model with independent rise and decay time constants [45] to calculate the dimensionless quantity, *g*(*t*). Dimensions of conductance are assigned by multiplying *g*(*t*) by ${\bar{g}}_{\text{synapse}}$. We write this model as two coupled differential equations:

$$\frac{dg(t)}{dt}=-\frac{g(t)}{\tau_{d}}+x\left(t\right),\;\tau_{\text{rise}}\frac{dx(t)}{dt}=-x\left(t\right)+r\left(t\right),$$ (8)

where *τ*_d_ is the decay time constant, *τ*_rise_ is the rise time constant, *x*(*t*) is the intermediate variable introduced to allow calculation of the second-order response of *g*(*t*) and *r*(*t*) is the stochastic process representing the total number of vesicles released by the presynaptic neuron at each point in time in a particular simulation, from its *N*_*M*_ release sites. Hence, *r*(*t*) ∈ {0, 1, …, *N*_*M*_}.

We considered two cases of the rise time: first, the more realistic case where the rise time was 0.1 ms (accounting for neurotransmitter diffusion, binding to postsynaptic receptors and receptor opening); second the case corresponding to the ‘delta synapse’ model, where *τ*_rise_ = 0 (a commonly used approximation to the finite rise time case). The latter case obviously corresponds to *x*(*t*) = *r*(*t*), with the result that only the first of the two differential equations in Eq (8) needs to be solved.

The peak synaptic conductance, ${\bar{g}}_{\text{synapse}}$, is determined by the quantal size, *q*, of a vesicle. In our simulations, the peak conductance at a release site depends on the presynaptic configuration. It was set for each simulation to meet the following criteria:

- *M* = 1 (“giant” case): depression at the maximum pre-synaptic firing rate of 50 Hz must be sufficient to move the mean population EPSP well below firing threshold, while depression at the minimum pre-synaptic firing rate of 10 Hz should leave the mean population EPSP near or above firing threshold;
- *M* = 512 (“cortical” case): temporal summation of multiple EPSPs is needed to reach firing threshold, while the mean postsynaptic rate at 10 and 50 Hz should be below saturation, so that there is a clear difference in postsynaptic firing rate between these frequencies.
- All *M*: similar relatively low mean post-synaptic firing rates, between about 5 and 25 spikes per second.

The chosen values are listed in Table 2.

### Overall experiments

Simulation runs with particular synaptic configurations and modulation frequencies between 0.1 and 5 Hz were repeated with the same input set of *M* pre-synaptic spike trains, but with different random seeds, resulting in different vesicle release patterns for each repeat, and in turn spike trains in the output neuron. Runs were also repeated up to 100 times with completely different sets of input spike trains to the same synaptic configuration. Simulation durations continued for up to 23 cycles of input modulation, and consequently the total simulation duration was inversely proportional to the modulation frequency. Data from the first three cycles were discarded for the purposes of calculating post-synaptic phase shifts, since at the start of the simulation all vesicle sites are assumed to be filled.

Table 3 summarises the parameter values we used that are common to all numerical experiments described in Results, other than those already listed in Tables 1 and 2.

**Table 3:** Summary of parameter values used for data shown in Results.

Parameter	Notation	Value	Units
Total number of release sites	*N*	512	N/A
Mean pre-synaptic spike rate	*A*	30	Hz
Peak pre-synaptic modulation	*B*	20	Hz
Vesicle replenishment time constant	*τ*_rec_	0.5	s
Vesicle release probability	*p*_v_	0.25	N/A

### Sinusoidally modulated input spike trains

Input spike trains are generated as independent, inhomogeneous Poisson processes. Spike times in individual trains are produced by the thinning method for generating inhomogeneous Poisson series [46], modified slightly so that each generated spike is followed by a refractory time of 2 ms during which no spikes can occur. Thus spike trains to different active zones have the same time-varying mean frequency but individual spikes are uncorrelated in time. In consequence, the “cortical” configuration is a completely uncorrelated pathway in which each of the 512 release sites is driven by an independent presynaptic spike train. In contrast, the “giant” configuration is a completely correlated pathway in which all 512 releasable sites may release independently, but are driven by spikes from a single presynaptic cell.

### Stochastic simulation of discrete vesicle release

The conceptual model of vesicle release site occupancy described above is implemented in the standard way [39], as a discrete-time, stochastic, finite-state automaton. At a particular time point *t*, a release site may be occupied by a releasable vesicle, or may be empty. An occupied site may release its vesicle with probability *P*_v_ on arrival of a presynaptic spike. One method for implementing this model is to simulate an empty site being refilled during the small time interval Δ*t* between time points *t* and *t* + Δ*t*, with probability Δ*t*/*τ*_rec_. However, we used a more efficient algorithm [39] for generating the stochastic process *r*(*t*), i.e. the total number of vesicles released at time *t*:

Input: Set of pre-synaptic spike times

Set: NextAvailabilityTime = 0 for all release sites

Set: r(t) = 0

For each active zone, j = 1:M

 For each pre-synaptic spike this active zone, occurring at time t_i

  For each release site this active zone, k = 1:N

   if t_i > = NextAvailabilityTime(j, k)

    //vesicle is available

    if Pr given_a (t_i) > unifrand()

     //Release the vesicle

     Set: NextAvailabilityTime(j, k) = t_i + exprnd()

     Set: r(t) = r(t) + 1

    end

   end

  end

 end

end

The function exprnd(⋅) generates exponentially distributed random numbers with a mean value of *τ*_rec_, and the function unifrand(⋅) generates uniformly distributed random numbers.

### Simulating the post-synaptic neuron’s membrane potential

We solved Eqs (2)–(6), with initial conditions *v*(0) = −66 mV, *m*(0) = 0, *h*(0) = 0 and *n*(0) = 0, using the Euler method. The time step we used, *t*_*s*_, was determined by the frequency of sinusoidal modulation. For *f* ≤ 1 Hz, we used *t*_*s*_ = 0.05 ms; for *f* ≥ 1 Hz we used a shorter time step of *t*_*s*_ = 0.05/*f* ms. Hence, with the maximum *f* of 5 Hz, the smallest time step size was 0.01 ms.

### Detecting and binning output spikes

In order to estimate the time-dependent spike-rates for the case of simulated HH model output neurons, we define the times of an output spike as the time instants, *t*_spike_, at which *v*(*t*_spike_ − *dt*) < 10 mV and *v*(*t*_spike_) > 10 mV. Although we use random synaptic input and the membrane potential fluctuates, we found that defining the spike threshold as 10 mV was sufficiently long into the upstroke of an action potential that we never observed a rapid reduction below 10 mV following its crossing, except in the actual downstroke. For the LIF model, the spike times are all time steps at which *v*(*t*) > *v*_thresh_.

Postsynaptic cell spike times were collected and collated from all repeats of all runs with a particular synaptic configuration. Spike time histograms were produced by binning in 5 ms time bins for the entire duration of each simulation other than the first three cycles of the modulation frequency.

### Quantifying phase shifts

To quantify the phase shift in the output spike train relative to the frequency of modulation of the input spike train, we first formed peristimulus time histograms (PSTH) for all simulations, by binning post-synaptic spikes into time intervals of duration *t*_b_ = 5 ms. We denote the total number of postsynaptic spikes in each of *K* bins as *r*_*k*_, where *k* ∈ {1, …, *K*} is the index of the *k*–th bin. For each value of *f*, the total phase since the beginning of a simulation for each such bin for an entire simulation was quantized, and we formed a polar coordinates representation for each bin given by

$$z_{k}=r_{k}\exp(i2\pi ft_{b}k).$$

This representation was then averaged over all bins (the input modulation is always present for an integer-number of cycles), and we use the resulting average phase value, $\bar{\phi }$. In other words, we obtained $\bar{\phi }$ that arises from

$$\bar{r}\exp\left(i\bar{\phi }\right)=\frac{1}{K}\sum_{k=1}^{K}r_{k}\exp(i2\pi ft_{b}k).$$

Note that this formula is equivalent to the “order parameter” in synchronized oscillation models, such as the Kuramoto model [47]. In that context, $\bar{r}$ is the phase coherence but we are not concerned with this entity here.

Note that since our simulations commence with no modulation, and then modulation proceeds starting from a phase of *π*/2 radians so that modulation is not discontinuous, the phase lead of the post-synaptic neuron is given by

$$\begin{array}{c} \phi_{l}=\pi /2-\bar{\phi }\;\text{radians}. \end{array}$$ (9)

## Results

### Synaptic depression and synaptic configuration interact to cause phase shifts

**Fig 2:**
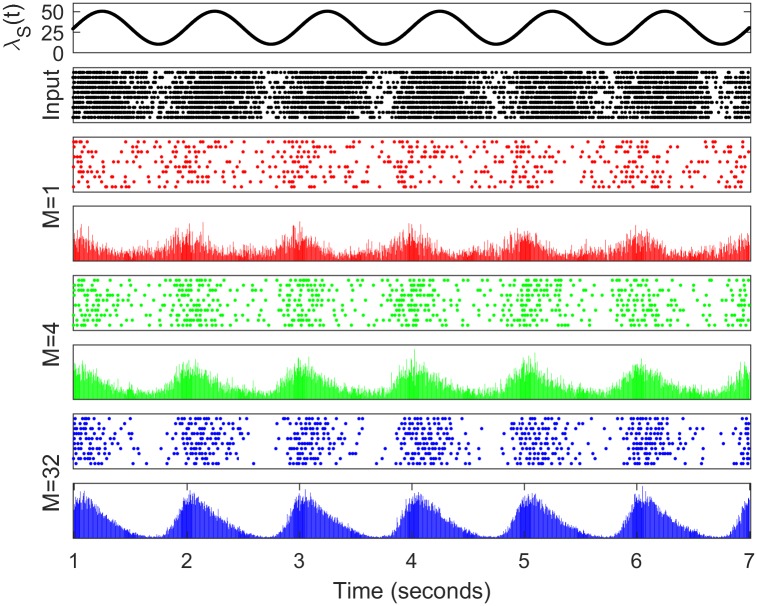
Raster plots and time-dependent histograms of simulated postsynaptic spiking of the model, in response to *f* = 1 Hz modulated pre-synaptic population inputs for different synaptic configurations with depressing synapses. The first panel shows the pre-synaptic spike-rate modulation, λ_S_(*t*). The second panel shows the spike times of 10 independently generated pre-synaptic spike trains with time-dependent inhomogenous Poisson rate λ_S_(*t*). The third, fifth and seventh panels show 10 post-synaptic spike responses for three distinct synaptic configurations (*M* = 1, *M* = 4 and *M* = 32), in response to 10 sets of independent pre-synaptic spike-trains. The fourth, sixth and eighth panels show histograms of the number of post-synaptic spikes as a function of time, in 5 ms bins, from 100 independent input spike-trains, and 100 independent trials for the vesicle releases for each input spike-train.

**Fig 3:**
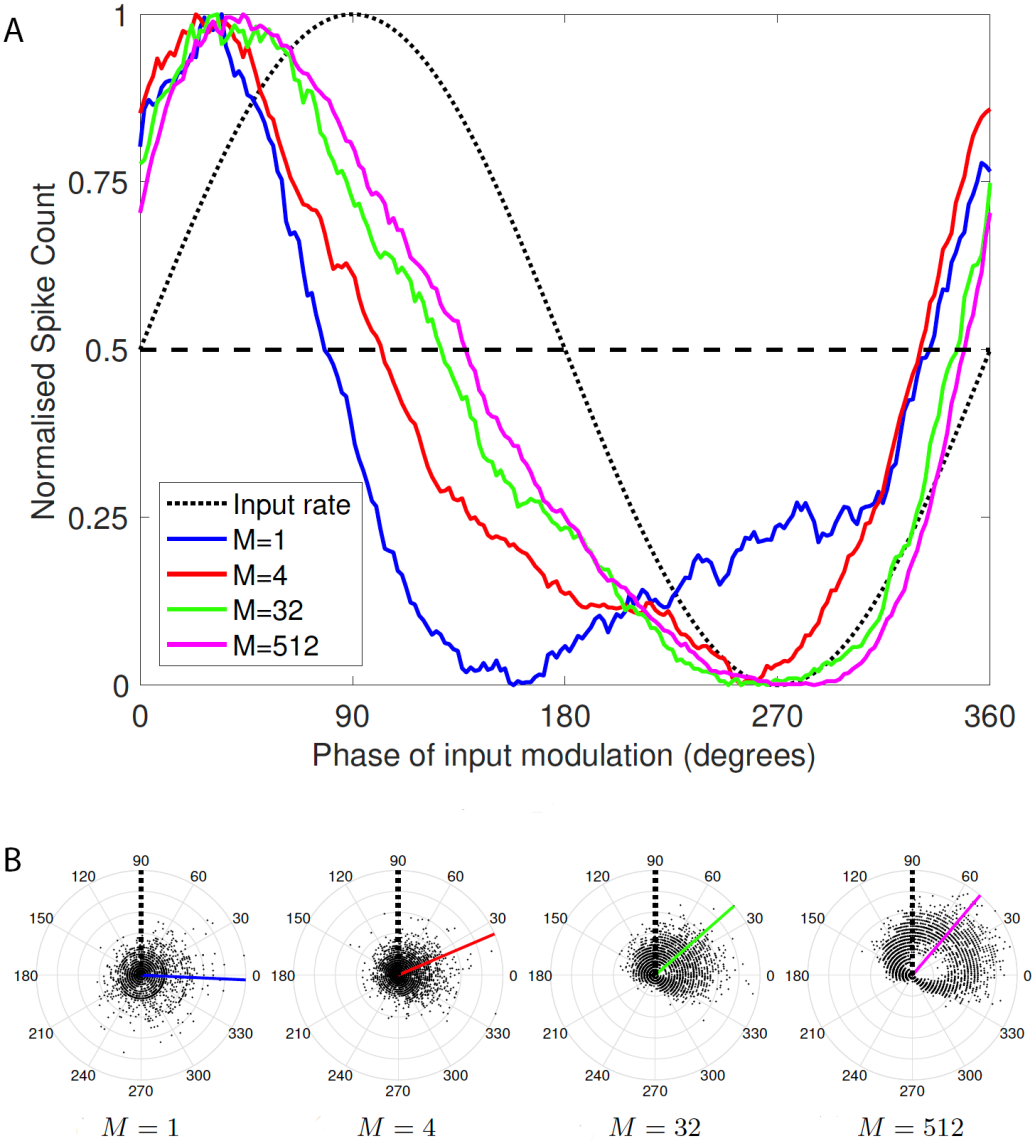
The phase of output spiking depends on synaptic configuration. The traces in Subfigure A were produced by forming histograms of postsynaptic spiking in response to 1 Hz-modulated population inputs for different synaptic configurations with depressing synapses. Spike times from each cycle of the input modulation in all cells in all runs with a particular synaptic configuration were binned in 1.8° phase bins. The histograms were then smoothed using a 20 sample moving average, and normalised. The dotted line shows the phase of the input modulation; since the modulation is defined as a sine wave rather than a cosine wave, the phase of its peak is at 90°, whereas the output spiking peaks at an earlier phase, and therefore has a *phase lead*. The dashed line makes it clear that the largest output phase lead (the blue trace crosses the dashed line at approximately 90° to the left of where the input modulation crosses the dashed line) is for *M* = 1 and the smallest (approximately 40°) for *M* = 512. Subfigure B shows scatter plots of each pair of phase and normalised spike count used to form the data shown in subfigure A; colored lines indicate the mean phase, in good correspondence with the crossings of the dashed line shown in A. The dotted black line shows that the phase of the input modulation is 90°, as in subfigure A.

**Fig 4:**
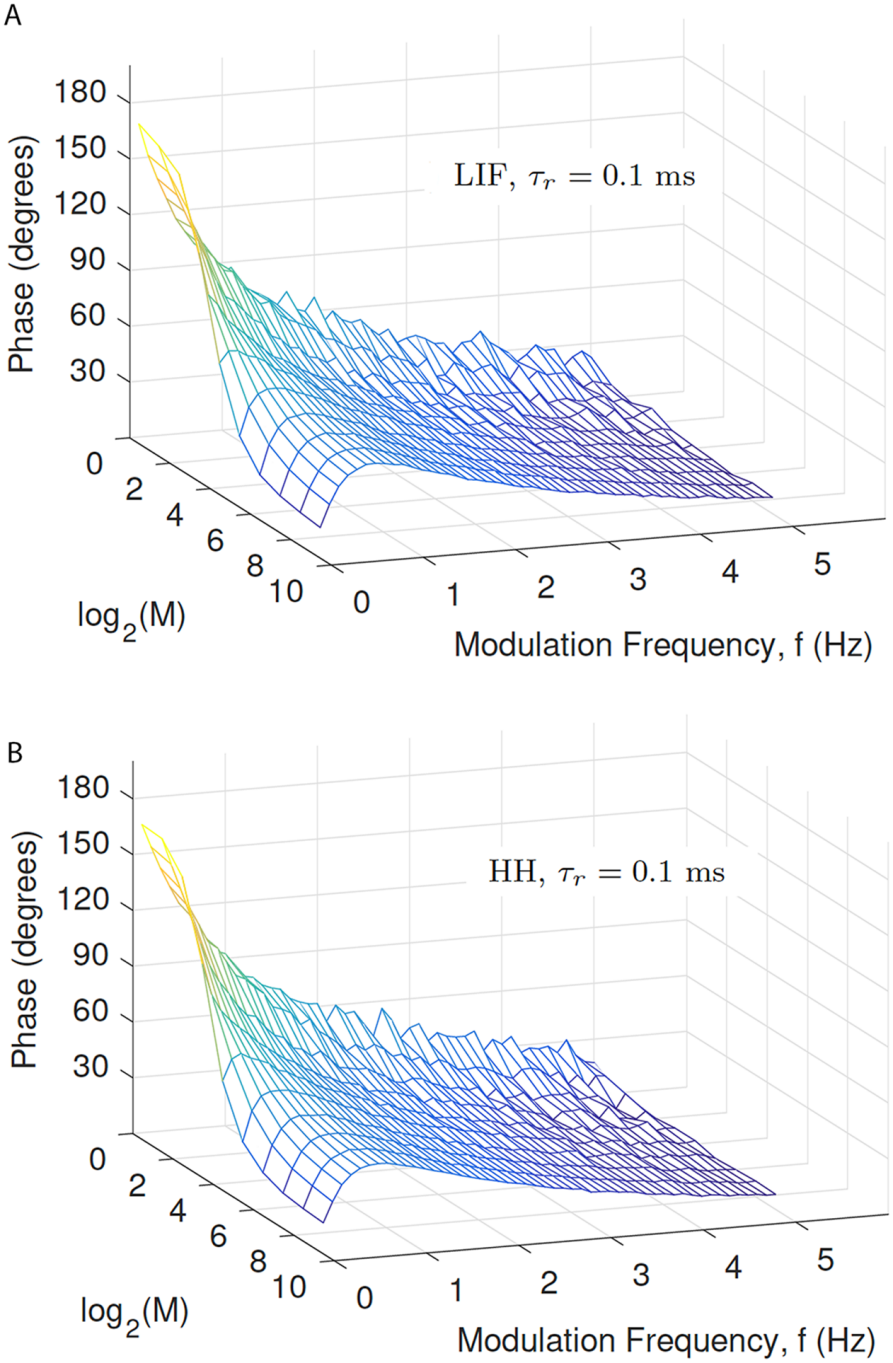
Phase lead of output spiking responses, relative to the periodic modulation of Poisson pre-synaptic spiking. Data is for both the LIF and Hodgkin-Huxley models, each with a rise time of 0.1 ms. As shown in S1 Fig, the phase change as a function of modulation frequency and the number of pre-synaptic neurons, *M*, is very similar when the post-synaptic conductance change has no rise-time.

Firstly we illustrate the interaction between synaptic depression and synaptic configuration on the phase of postsynaptic spiking relative to a sinusoidally-modulated input driving frequency. As an example, we simulated the HH model using modulation frequency *f* = 1 Hz and show resulting spike rasters for 10 independent input spike trains in Fig 2. Time-dependent histograms formed from many more repeats are also shown in Fig 2. We observe from these plots that the peak spiking response via the giant synapse configuration (*M* = 1) is largely out of phase with the input modulation, whereas for synaptic configurations with more, but smaller active zones, the response is closer to being in phase, but still leads the input. These phase shifts are quantified and more clearly illustrated in Figs 3 and 4. To verify the significance of our claim that phase depends on synaptic configuration, we applied the pair-wise t-test to the phases of binned simulation data for *M* = 1 compared with the phases for *M* = 2, 4, 8, …, 512 (as plotted in Fig 3(B) for *M* = 1, 4, 32 and 512). We found that for all *M* greater than 4, the null hypothesis that the phase distributions had equal means can be rejected at the 5% level. For *M* = 4, the p-value was 0.0019 and for *M* = 8, the p-value was 2.6 × 10^−5^. The p-value decreased rapidly for larger *M*.

**Fig 5:**
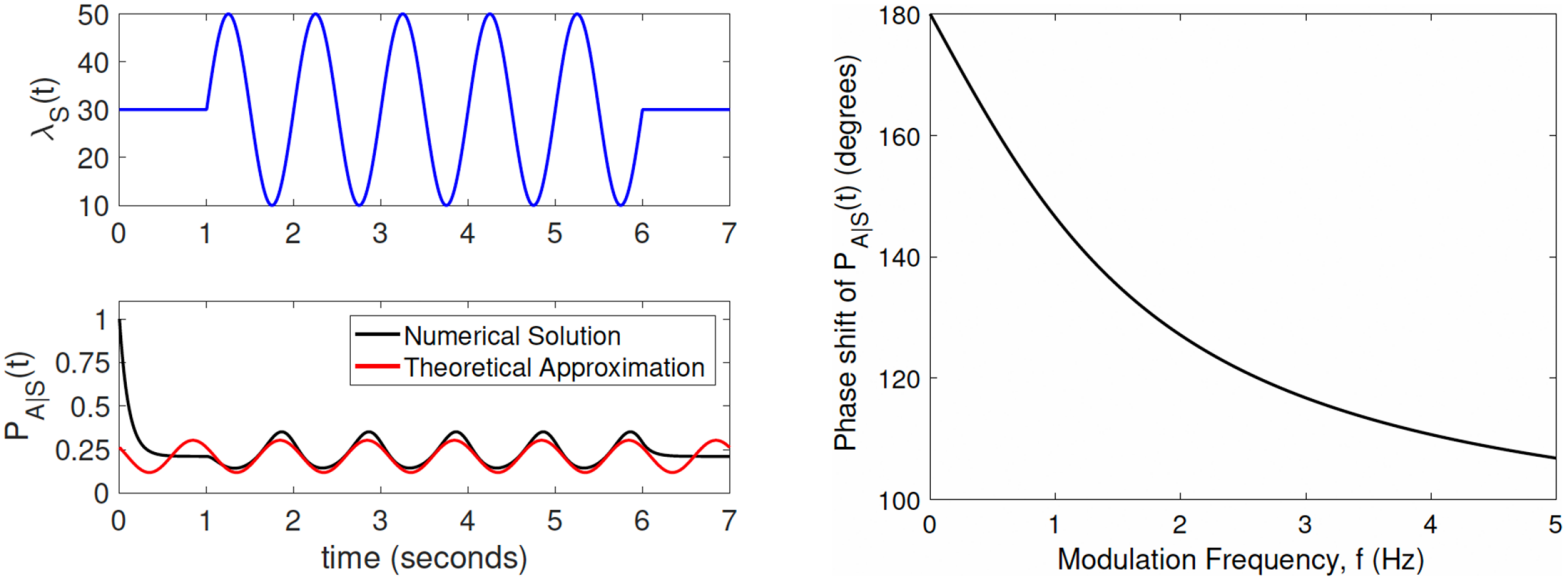
Theoretical calculations of phase shift. The lower left panel shows that for a modulation frequency of *f* = 1 Hz, our mathematical derivation of an approximate steady state solution to Eq (10) correctly obtains the phase shift in the conditional vesicle availability probability, *P*_A|S_(*t*), relative to periodic input spike rate modulation, λ_S_(*t*). In the legend, ‘Theoretical’ corresponds to Eq (12). Parameter values are as shown in Table 3. The right-side panel is shown to make it clear that there is a large phase shift relative to the input stimulation. The phase shift according to a numerical integration of Eq (21) is 144.54° for the numerical solution and 146.52° for the theoretical solutions. The right panel shows the theoretically calculated phase shift (Eq (13)) of conditional vesicle availability probability, *P*_A|S_(*t*). The phase shift is relative to periodic input spike rate modulation, λ_S_(*t*), as a function of modulation frequency, *f*.

These phase changes can be explained as follows. As we show below mathematically (see also Fig 5) the release probability (which is the product of vesicle availability and individual vesicle release probability) at an individual release site is strongly out of phase with the input signal frequency modulation, so that it increases as the signal frequency decreases, and vice versa. It is well known for a depressing synapse that the probability of release is approximately inversely proportional to the driving frequency at high frequencies [12]. Consequently, in the “cortical” configuration of *M* = 512 independent AZs, the mean synaptic current is proportional to the driving frequency multiplied by the release probability, and so is only weakly dependent on frequency at high frequencies. For the frequencies used here (10 to 50 Hz), a small dependency does remain so that the synaptic current follows the modulated driving frequency. However, as the input frequency gradually increases from low to high (at the modulation frequency), depression also increases gradually but faster than the input firing rate, leading to the summed synaptic current peaking before the peak of the driving frequency.

In the case of the “giant synapse” (*M* = 1) configuration there is no temporal summation of EPSPs across release sites and the mean synaptic current no longer determines output spiking. Instead, the population of sites releases synchronously on arrival of each presynaptic action potential. The amplitude of the consequent EPSC is thus directly proportional to the release probability, which, in turn, is an inverse function of the input frequency. In the spiking regime for this configuration, as the input frequency increases, EPSCs depress to the point at which a population EPSC no longer drives the output to spiking threshold. On the other hand, as the input frequency decreases, output spiking becomes more reliable as the population EPSC amplitude increases. The spike histogram obtained over a population of cells does not exhibit a perfectly out-of-phase response (see Figs 2 and 3), compared to the input signal. Though the population EPSC is largest at the lowest input frequency, the rate of output firing is highest as the input frequency increases but the population EPSC is still large enough to cause postsynaptic spikes. Strictly speaking, the population response shown in Figs 2 and 3 for *M* = 1 exhibits a very large phase lag, since the output cells do not respond at all to the initial peak in input frequency, with the first large response occurring on the upward slope of the second input modulation cycle. However, given that this response is signalling the start of this new cycle, for continuous modulation it seems more appropriate to deem this a functional phase lead.

For synaptic configurations between the two extremes, the response shifts smoothly, but quite rapidly from the spatial summation of the giant synapse to the temporal summation of the cortical synapse. Thus the phase lead reduces quickly as the number of active zones driven by independent spike trains increases (see Fig 3). So a configuration even with only *M* = 8 AZs (see Fig 4) is largely dominated by temporal summation and acts much like the extreme cortical case, though still with a larger phase lead. The lead is almost at the extreme cortical level with *M* = 32 AZs (Fig 3A).

### Phase-shifts depend on modulation frequency but not post-synaptic spiking dynamics

The observations presented above were all for a modulation frequency of *f* = 1 Hz and the HH model. However, we found that the frequency of modulation significantly affected the phase-shift, but that the post-synaptic spiking dynamics did not. To illustrate these findings, Fig 4 and S1 Fig shows the phase shift as a function of both the number of active zones *M*, and modulation frequency, *f*, where *f* ∈ [0.1, 5] Hz.

It is clear from these figures that the patterns of phase shift are not significantly different for the HH and LIF models. In both cases, the phase shift generally decreases with increasing modulation frequency, and with increasing number of AZs, *M*. Output spike rates are also similar across models (S2 Fig).

An interesting exception to this trend that holds for both models, is the case where *M* is large (the “cortical” configuration) and *f* is small. In this case the phase shift has a local maximum near *f* = 1 Hz. This feature has the attributes of a resonance phenomenon. Later in this paper, we show using theoretical approximations, that the frequency of the resonance for large *M* can be predicted by the value of the longest time constant in the model, namely the replenishment time constant, *τ*_rec_, and also by the vesicle release probability, *P*_*v*_, which together with *τ*_rec_ determines the time course of depression.

The pre-synaptic time constants mean that the output spiking response varies with modulation frequency, as this interacts with the time course of depression. For the giant synapse (*M* = 1) with very slow modulation (*f* = 0.1 Hz), the output response is nearly completely out of phase with the input. With such a slow modulation, depression and recovery have time to follow the input frequency. As the frequency increases, depression drives the EPSC amplitude below the spiking threshold for the postsynaptic cell. Conversely, recovery from depression as the input frequency decreases increases the EPSC amplitude and the output cell spikes in correspondence with the lowest input frequencies. However, at higher modulation frequencies, output spiking increasingly occurs on the rising input phase before depression has a chance to progress. So the phase lead of the output decreases as the peak of output spiking is shifted towards the peak of input spiking. These effects occur when there is minimal temporal integration of EPSCs between input stimuli. If the postsynaptic EPSC decay time is long, then the strongly out-of-phase response for *M* = 1 disappears and the response is more like the cortical configuration (S3 Fig).

For the cortical configuration (*M* = 512), the output response exhibits a small phase lead at all frequencies, however the largest lead is at *f* = 1 Hz. At low modulation frequencies (e.g. *f* = 0.1 Hz), the depression level follows the input frequency. Depression is maximal at an instantaneous input rate of 50 Hz, but this is still insufficient to eliminate an increase in output firing with the increase in driving frequency. Thus the output response essentially tracks the input. At high modulation frequencies (a few Hertz), depression is little modulated by changes in the input frequency, again resulting in the output largely tracking the input. However, at around *f* = 1 Hz, depression has time to increase as the firing frequency increases, but the increase in the input frequency is sufficiently fast that the onset response before the synapses depress dominates the output spiking, resulting in a significant phase lead (but still small compared to the giant synapse configuration).

### Mathematical analysis predicts phase shifts observed in our simulations

We now show that the central results found in our simulations can be predicted mathematically from the model.

#### Phase shifts in vesicle availability

We introduce *P*_A|S_(*t*) as the conditional probability of vesicle availability at time *t*, given a spike arrived at time *t*. Recall that the probability of release of an available vesicle upon a pre-synaptic spike arrival is *P*_v_ and the time-constant of refill is *τ*_rec_. As studied by [48], given a pre-synaptic spike occurs at time *t* in a pre-synaptic neuron, the probability of availability of a vesicle at any arbitrary release site on the pre-synaptic neuron is the solution to

$$\frac{dP_{A|S}\left(t\right)}{dt}=\frac{1-P_{A|S}\left(t\right)}{\tau_{\text{rec}}}-P_{v}\lambda_{S}\left(t\right)P_{A|S}\left(t\right).$$ (10)

This equation captures that the rate of change in vesicle availability is given by stepwise decrements proportional to the probability of successful release, triggered upon arrival of each action potential (rightmost term on right hand side), followed by exponential recovery (leftmost term on right hand side).

An example of a numerical solution of this equation is shown in Fig 5 (second panel, black trace).

Eq (10) has a known integral-form solution. For the case we are interested in where λ_S_(*t*) has the sinusoidal form of Eq (1), the solution has a transient term that quickly decays and a steady state solution we can write as

$$P_{A|S}\left(t\right)∼\frac{1}{\tau_{\text{rec}}}\exp(\gamma \cos\left(\omega t\right))\int_{0}^{t}\exp\left(-(t-\theta )/\kappa \right)\exp(-\gamma \cos\left(\omega \theta \right))d\theta ,$$ (11)

where *ω* ≔ 2*πf*, *κ* ≔ 1/(1/*τ*_rec_ + *P*_v_ *A*) and $\gamma ≔\frac{BP_{v}}{\omega }$. Substituting the approximation exp(±*γ*cos(*ωθ*)) = 1 ± *γ*cos(*ωθ*) into the integral in Eq (11) and simplifying, results in the approximate solution

$$P_{\text{A}|\text{S}}(t)\sim \frac{\kappa }{\tau_{\text{rec}}}\left(1+\frac{BP_{\text{v}}\kappa }{\sqrt{1+\omega^{2}\kappa^{2}}}\text{cos}(\omega t+\text{arctan}(1/(\omega \kappa )))\right).$$ (12)

Hence, relative to λ_S_(*t*), the overall phase shift of *P*_A|S_(*t*) is *π* − arctan(*ωκ*), i.e. the phase shift is

$$\begin{array}{c} \Theta ∼\pi -\arctan(\frac{\tau_{\text{rec}}\omega }{1+\tau_{\text{rec}}P_{v}A})\;\text{radians}. \end{array}$$ (13)

As the modulation frequency approaches 0, this phase shift approaches *π*, while *P*_A|S_(*t*) approaches a constant value of $\frac{\kappa }{\tau_{\text{rec}}}=\frac{1}{1+\tau_{\text{rec}}P_{v}A}$. As shown in Fig 5(B), the phase shift decreases from this case as modulation frequency increases, down to about 108 degrees for the maximum modulation frequency considered of 5 Hz.

As illustrated in Fig 5A, the approximation of Eq (12) may be inaccurate in terms of the mean value relative to the numerical solution of Eq (10) but the phase of the sinusoidal term is the same in both cases. The accuracy of this phase approximation depends on the specific parameter values, but as shown in Fig 5, it is very accurate for *f* = 1 Hz and the parameter values used in this paper.

Regardless of the accuracy for all parameter values, the main conclusion from this analysis is that the vesicle availability probability is modulated periodically, and has a frequency-dependent phase shift relative to the input-spike rate modulation. This conclusion is consistent with the frequency dependence of the phase shifts found in our simulations, as illustrated in Fig 4.

#### Phase shifts in pre-synaptic release rate

Our simulations revealed that when the number of presynaptic neurons *M* is small, the phase of the time-dependent spike-rate of the post-synaptic neuron follows quite closely to the phase offset of the vesicle availability, but the spiking phase offset has a significant dependence on the synaptic configuration. An intuitive explanation for this effect is as follows.

For the calyx case, (a single pre-synaptic neuron, *M* = 1), up to *N* vesicles can be released simultaneously in the model. Provided the post-synaptic neuron is not in its refractory period, a sufficiently large number of vesicles released simultaneously will cause the post-synaptic neuron to spike very rapidly after such an event. This is in contrast with the cortical case of large *M*; in this configuration, vesicle release is not simultaneous, and the post synaptic neuron needs to integrate synaptic input over a longer time period before it will spike.

For *M* = 1 we found that the post-synaptic neuron model we use is most likely to spike at phases in which the number of available vesicles is large, because this is when the most number of vesicles will be released simultaneously. This is likely to follow closely in time the phase at which the input spike rate is low.

On the other hand, for large *M*, the post-synaptic neuron is most likely to spike at phases in which the average release rate is highest, because this is when the independent EPSCs will be most likely to integrate to a high enough value to cause a spike.

For the intermediate configurations, we have to take into account that multiple pre-synaptic neurons might combine to release enough vesicles within a short time period to cause the post-synaptic neuron to spike. To account for this general case, we first consider the time-dependent total expected number of pre-synaptic spike-arrivals to the *M* pre-synaptic neurons during a time-period of length *τ*_p_. This is

$$\begin{array}{c} a\left(t\right)≔M\int_{t-\tau_{p}/2}^{t+\tau_{p}/2}\lambda_{S}\left(\theta \right)d\theta . \end{array}$$ (14)

We assume that *τ*_p_ is small compared to 1/*f* (*f* is the frequency of modulation), and therefore that we can approximate this integral as

$$\begin{array}{c} a\left(t\right)≃M\tau_{p}\lambda_{S}\left(t\right). \end{array}$$ (15)

However, *a*(*t*) is an average, while in any realisation of the model, an integer number of pre-synaptic spikes arrives during time *τ*_p_. Hence, to obtain an approximate expression for the random variable describing the actual number of pre-synaptic spike arrivals, we quantize this average, using the ceiling operator, ⌈⋅⌉ to get

$$\begin{array}{c} b\left(t\right)≔\lceil M\tau_{p}\lambda_{S}\left(t\right)\rceil \;\;\in \left\{1,2,3,\cdots \right\}. \end{array}$$ (16)

For the minimum value of *b*(*t*) of 1 spike to be in agreement with the underlying model in the extreme case of *M* = 1, we require *τ*_p_λ_S_(*t*) ∼ 1. With the parameters we use, λ_S_(*t*) ∈ [10, 50]. Since we are interested in phase-shift, it suffices for our approximation to be valid only at some point during a cycle of the pre-synaptic modulation, and hence we need only ensure *τ*_p_ ∼ 1/ max(λ_S_(*t*)) = 20 ms to have an expectation of at least one spike at some point in a cycle.

Based on these insights, we find the following approximation for the post-synaptic spike rate, *c*(*t*). We begin by noting that the time-averaged probability of any release site releasing a vesicle is *P*_v_*P*_A|S_(*t*) and hence the expected number released at an active zone is *N*_M_ *P*_v_ *P*_A|S_(*t*). We then combine this with Eq (16) to obtain

$$\begin{array}{ll} c\left(t\right) & =N_{M}P_{\text{v}}P_{\text{A}|\text{S}}\left(t\right)b\left(t\right)\\ & =N_{M}P_{\text{v}}P_{\text{A}|\text{S}}\left(t\right)\left\lceil \tau_{\text{p}}M\lambda_{\text{S}}\left(t\right)\right\rceil . \end{array}$$ (17)

Note that without the ceiling operator, *c*(*t*) is identical for all configurations in our model, since *N*_*M*_ = *N*/*M*. The ceiling operator models the fact that a presynaptic spike during each interval of duration *τ*_p_ is a binary event—it either occurs or doesn’t occur; by extension, it models the binary event that either sufficient or insufficient vesicles are released during *τ*_p_ to cause a post-synaptic spike. This all-or-nothing aspect is crucial when *M* is small, since in this regime the average does not characterise the system.

Put another way, we have a system which can act as both an integrator (cortical case) or a coincidence detector (calyx case), and span the intermediate regimes between these two extremes, depending on the synaptic configuration. Previous modelling on stochastic short term plasticity identified that pairing different kinds of pre-synaptic and post-synaptic spiking mechanisms can also lead to integration or coincidence detection [49], but in contrast here the effect is due to connectivity and not dynamics.

Assuming *c*(*t*) is periodic, we can estimate its phase offset relative to λ_S_(*t*) using the first-order complex Fourier series representation of *c*(*t*). That is, we denote the phase offset as *ϕ* = arg[*z* exp (i*ϕ*)] and calculate

$$z\exp\left(i\phi \right)=\int_{0}^{T}c\left(t\right)\exp(i\frac{2\pi t}{T})dt,$$

where $i≔\sqrt{-1}$, and *T* is the period of λ_S_(*t*).

Hence, we can approximate the phase shift theoretically by numerically calculating

$$\Theta (M,f)=\text{arg}[\int_{0}^{\left(1/f\right)}P_{A|S}\left(t\right)\left\lceil \tau_{p}M\lambda_{S}\left(t\right)\right\rceil \exp(i2\pi ft)dt]$$ (18)

$$\begin{array}{cc} & ≃\text{arg}[\int_{0}^{\left(1/f\right)}\frac{BP_{v}\kappa }{\sqrt{1+\omega^{2}\kappa^{2}}}\cos\left(\omega t+\arctan\left(1/(\omega \kappa )\right)\right)\left\lceil \tau_{p}M\lambda_{S}\left(t\right)\right\rceil \exp(i2\pi ft)dt] \end{array}$$ (19)

**Fig 6:**
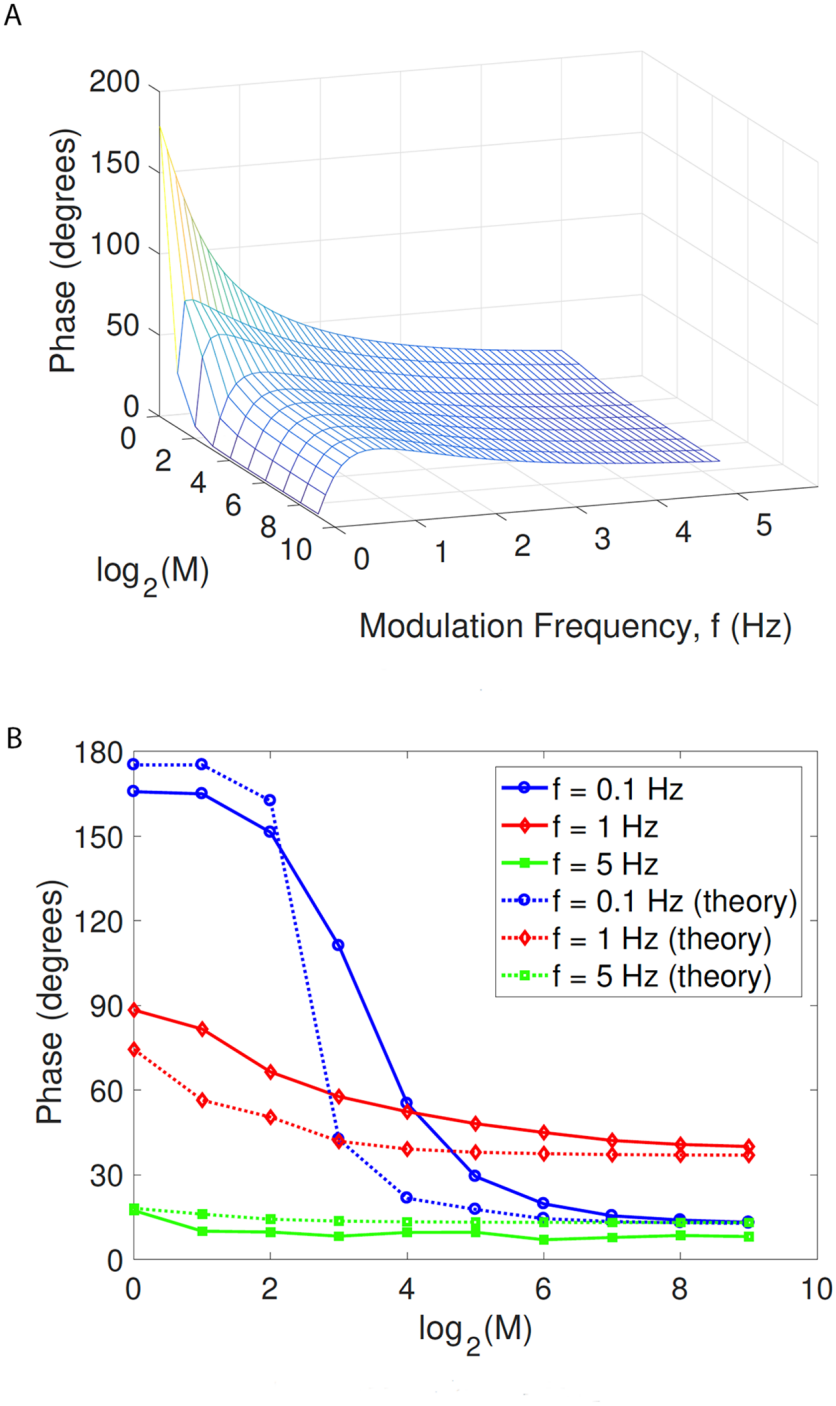
Theoretical approximation of Eq (18). Subfigure A, obtained with *τ*_*p*_ = 30 ms, shows that our theoretical prediction of the phase shift is strongly in qualitative agreement in terms of the trend with *M* and *f* obtained from simulations and shown in Fig 4. Subfigure B shows that the quantitative agreement is superior for larger values of *M* and *f*; here we have adjusted *τ*_*p*_ independently for each value of *f*, namely *τ*_p_ = 10 ms for *f* = 0.1 Hz, *τ*_p_ = 30 ms for *f* = 1 Hz, and *τ*_p_ = 60 ms for *f* = 5 Hz.

Fig 6 shows the result of calculating Eq (18) as a function of *f* and *M*. Clearly the trends in the phase shift from this approximation strongly resemble the trends in phase shift as *M* and *f* vary as obtained from simulations and shown in Fig 4. In particular, the data predicts the resonance in the phase lead observed using simulations of the cortical case, i.e. as seen in Fig 4.

#### Dependence of phase shifts on time-constant of depression and vesicle release probability

**Fig 7:**
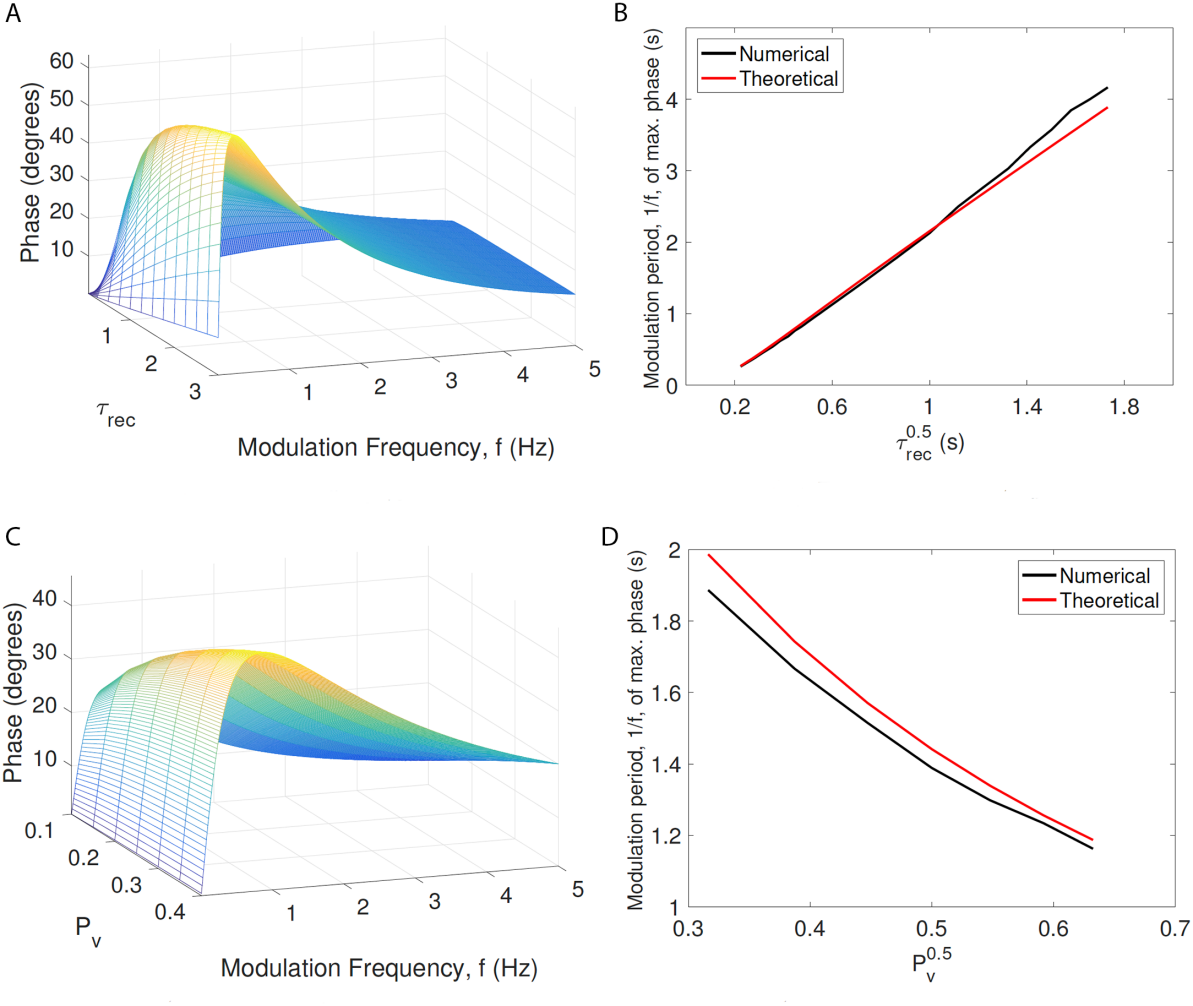
Theoretical analysis of resonance in phase change vs system dynamics. Subfigures A and C show, for *M* = 512, that the mathematically predicted resonance in the phase change observed in Fig 6A depends strongly on the longest time constant in the system, *τ*_rec_ and on the vesicle release probability, *P*_*v*_. Subfigures B and D show, for *M* = 512, that the period of the resonance has an approximately linear relationship with $\sqrt{\tau_{\text{rec}}}$ and $\sqrt{P_{v}}$. Together, *τ*_rec_ and *P*_*v*_ determine the time course of depression, which determines phase.

Finally, we investigated the relationship between the phase-shifts observed and the time constants in the model. Firstly, Fig 7A shows how our theory predicts the phase shift will vary as the time constant of depression, *τ*_rec_, and the modulation frequency, *f*, are varied. The data shows that the resonance in the phase lead persists as *τ*_rec_ increases.

To investigate this effect further, we examined how the modulation frequency at which the resonance occurs varies with *τ*_rec_. Fig 7B indicates that the period of the resonant frequency has an approximately linear relationship with the square root of the time constant of depression in the model, *τ*_rec_.

This observation can be verified mathematically as follows. We first note that for the cortical case of large *M*, that the ceiling operator used in Eq (21) can be removed; for this case, we expect that the phase will be determined from the product

$$\hat{c}\left(t\right)≔P_{A|S}\left(t\right)\lambda_{S}\left(t\right).$$ (20)

Under this assumption and using the approximation of Eq (12), the phase shift in pre-synaptic spike rate can then be calculated in a similar manner to the calculation of phase in Eq (12), by multiplying Eq (12) by Eq (1), expanding, and simplifying the terms at frequency *f*. The resulting difference in phase between *P*_A|S_(*t*) and $\hat{c}\left(t\right)$ can be simply expressed as arctan(*ωτ*_*a*_) − *π*.

This expression enables us to predict the frequency of the resonance for large *M*. We write the phase of $\hat{c}\left(t\right)$ as

$$\hat{\theta }=\arctan\left(\omega \tau_{a}\right)-\arctan\left(\omega \kappa \right)-0.5\pi .$$ (21)

This expression is maximised when $\omega =\frac{1}{\sqrt{\tau_{\text{rec}}\kappa }}$. For our chosen values in Table 3, this is equivalent to *f* ≃ 0.69, in excellent correspondence with our data.

Finally, we note that although *κ* depends on *τ*_rec_, the scaling of *ω* with *τ*_rec_ remains consistent with Fig 7B.

The quantity *κ* is equivalent to the time constant of depression following a step change in driving frequency, and this also depends on the vesicle release probability, *P*_*v*_. Carrying out a similar investigation by varying *P*_*v*_, we see that the resonant frequency of maximum phase change is also a near inverse linear function of the square root of *P*_*v*_ (Fig 7(c) and 7(d)), as is to be expected from the expression above.

## Discussion

### Model considerations

In this theoretical study we have demonstrated that short-term synaptic depression interacts with rhythmic inputs oscillating with a period in the same order as the time constant of recovery from depression, such that the phase of the output spiking response may differ significantly from the input. In particular, the output exhibits a phase lead. Further, the magnitude of this phase lead depends on the configuration of the input synaptic pathway. To demonstrate this we considered a synaptic pathway with a fixed number of release sites, each of which may be occupied by and release a single vesicle, independently of all other sites. These release sites each received a frequency-modulated Poisson spike train, but with variations in the correlations between these trains at different release sites. In a “cortical” configuration, all spike trains were independent. In this situation the postsynaptic cell is driven by the temporally integrated EPSCs from all sites and largely follows the firing rate of the inputs. However, interaction with the time constant of release site depression results in a small phase lead of the output firing: the postsynaptic response begins to decrease before the input reaches its peak firing rate, due to depression. At the other extreme of a “giant” synapse, all release sites are driven by the same spike train, but release vesicles independently. In this case the postsynaptic response is determined by individual presynaptic spikes: there is little temporal summation between spikes as all sites release synchronously with the arrival of a spike. So the postsynaptic response is strongest when population EPSCs are largest, which occurs at the lowest input firing rate when release sites are least depressed (i.e. most likely to contain a releasable vesicle). Thus the output firing is strongly out of phase with the modulation in input frequency.

We considered two models for the post-synaptic neuron (the HH model and the LIF model) and found that both models produced nearly the same results regardless of synaptic configuration, despite the HH model exhibiting Class-II excitability and the LIF model Class-I excitability. We also found that the presence of a 0.1 ms rise-time in the synaptic EPSC produced nearly identical results to a delta-synapse response (rise time of zero ms). These comparisons suggest the key parameters that determine how phase alters with configuration (i.e., how temporal integration declines as release becomes more synchronous) are the time constants involved—the synaptic decay-time, and the post-synaptic membrane time constant. Together, these time constants can be thought of as the “integration time”.

In the simulated synaptic pathways demonstrated here, there is a rapid transition from the spatial-summation-dominated to the temporal-summation-dominated regime, which takes place when the number of active zones increases from 8 to 16 (out of a possible 512; see Figs 3 and 4). Further, the temporal-summation-dominated regime (number of active zones above around 8) exhibits a maximum phase shift at a modulation frequency that is dependent on the depression time course (see Figs 4 and 7).

Many synaptic pathways exhibit other forms of short-term plasticity, including facilitation, augmentation, release-independent depression and activity-dependent recovery from depression [37, 50]. Initial simulation results [35] indicate that these mechanisms can significantly affect the phase shifts due to depression alone. Facilitation can increase the phase shift at large synapses by increasing release even at low stimulation frequencies, but decrease it in the cortical case (many small, independent active zones) by delaying the onset of depression. Facilitation and depression interact to give a preferred stimulation frequency for a synapse at which EPSCs are maximal. Modelling and analysis of a network of two neurons connected by reciprocally inhibitory synapses with mixed dynamics, shows that the relative combination of facilitation and depression can determine either the period or the relative phase of firing of the neurons [31]. Sufficiently strong activity-dependent recovery from depression can largely remove the phase shift by minimising depression. In this current paper we have concentrated on pure depression, since this is the underlying cause of the phase shift and the model is simple enough to allow the formulation of approximate theoretical expressions for the components determining the phase shift (vesicle availability and release).

### Biological significance

The impact of synaptic dynamics on the phase of a neuronal response is potentially significant in a wide range of neural subsystems, particularly for sensory processing and generating motor outputs, where signals may vary over similar time scales (tens of milliseconds to seconds) to STP mechanisms. However, this has not been studied directly in experiments, perhaps because it has not been recognised as a potential effect. It is also very difficult to experimentally alter specific STP mechanisms in a controlled way [51] to be able to quantify the effects of STP on the phase of a response. Here we discuss key neural systems where the demonstrated phase shifts could be significant.

One other theoretical study has shown the ability of short-term depression to produce phase advances to oscillating inputs [34]. Neuronal responses in visual cortex to spatially and temporally oscillating visual inputs are known from experiments to be highly non-linear to changes in features such as contrast. In [34], a spiking neural model driven by a population of synaptic inputs demonstrated that synaptic depression could be a significant component of such non-linearities, particularly changes in phase advance of the neuronal response. Here we have produced a more comprehensive picture of the effects of synaptic depression on phasic responses, using a more biophysical synapse model than employed by [34] and we have quantified the phase shifts relative to stimulus rhythms, STP dynamics and synaptic configuration.

Phase leads in the “cortical” configuration (see Fig 3) could be sufficient to compensate for synaptic transmission delays. It has been observed that inhibition leads excitation in visual cortical responses to light flashes [52], despite the extra neurons and synapses involved in the inhibitory response. Such compensation for delays could be particularly important for motor systems, such as the vestibular-ocular reflex (VOR). The VOR must maintain a very precise eye rotation that is equal-and-opposite to a head rotation, so that we can focus on an object despite head movement. Thus this circuit requires a gain of 1 and a response with minimal time delay. A suitable phase lead could provide the necessary compensation for transmission delays in components of this reflex circuitry. In fact, phase leads are seen in the responses of vestibular hair cells to head rotations [53], negating the need for further phase shifts at subsequent synaptic connections. Experimental recordings from the principal synaptic connection of this reflex, between the vestibular afferents and medial vestibular nucleus neurons, during physiological stimulation does not reveal significant STP [54], with minimal depression due to rapid replenishment of multiple low-probability vesicle release sites [55]. Computer simulations demonstrate that rapid, activity-dependent replenishment can indeed minimise phase shifts through such a pathway [35]. Thus it seems this synaptic connection is specifically designed to avoid further phase shifts in the VOR, beyond those produced by the electro-mechanics of the sensory receptors.

Phase in motor systems is particularly important for multi-motor-unit coordination during movement. Swimming in the lamprey requires generation of a constant phase delay between motor units along the length of the body to generate the required oscillating body pattern for swimming at different speeds. Various mechanisms for maintaining this phase delay have been proposed [33], but the contribution of STP so far has not been considered. Recent work has begun to experimentally characterise STP in these lamprey motor units and examine its effects in patterning network activity [51]. Depression determines the burst length and frequency of neuronal firing in this circuit in response to a non-physiological constant drive. Multi-unit coordination and phase delays were not studied. This work [51] highlights the difficulties with experimentally isolating the effects of STP in a network setting. Neurotransmitter release, and hence STP, is most easily adjusted by altering external calcium levels in tissue slice experiments, but changes in calcium can also affect neuronal membrane excitability. Specifically altering vesicle recycling (a determinant of depression) or release probability (e.g. facilitation) exclusively is not possible.

Giant synapses exist in nervous systems, particularly in pathways in which temporally precise and reliable responses are important. In the mammalian nervous system, the end bulb of Held and the calyx of Held are successive such synapses in the auditory brain stem [36]. Here, reliable and precise postsynaptic firing to inputs ranging up to several hundred Hertz is required. Ideally, these synapses would not exhibit STP, but both depressing and facilitating STP mechanisms are present and affect physiological responses. The postsynaptic neurons are capable of firing to even strongly depressed EPSPs, thus the anti-phase firing demonstrated here for such synapses is not evident, though could be revealed through, for example, an increase in inhibition of the postsynaptic neuron that effectively increased its firing threshold. However, the phasic preference for firing induced by STP could underpin the stronger phase locking of the output firing compared to the input spiking recorded *in vivo* from the calyx of Held pathway in the medial nucleus of the trapezoid body [56].

In conclusion, in addition to rate filtering, short-term synaptic depression affects the phase of neuronal responses to time-varying inputs. This is likely to be significant for many neural systems in which timing is critical, such as motor responses, and is a factor that needs to be considered when interpreting dynamic neural responses.

## Supporting information

S1 Fig Phase lead of output spiking responses for a synapse model without a rise time, relative to the periodic modulation of Poisson pre-synpatic spiking. The trend as a function of modulation frequency and the number of pre-synaptic neurons, *M*, is very similar whether the output neuron is a leaky integrate-and-fire (LIF) model or Hodgkin-Huxley (HH).
(TIFF)

S2 Fig Mean-spike rate of output spiking responses for different models. Unlike the phase lead, the spike rates observed and the manner in which they vary with modulation frequenecy and *M*, has a dependence on the model and whether the post-synaptic conductance change has a zero or non-zero rise-time.
(TIFF)

S3 Fig Phase and mean-spike rate of output spiking responses for a long synaptic time constant, *τ*_*d*_ = 10 ms. The model used is otherwise the same as the main text. The mean spike rate with such a long time constant becomes very large relative to *τ*_*d*_ = 1 ms, and the general trend of the phase now remains similar for all M due to increased temporal integration, even for low *M*.
(TIFF)
