# Supplementary figures and images for: Phase changes in neuronal postsynaptic spiking due to short term plasticity
Source: PLoS Comput Biol. 2017 Sep 22;13(9):e1005634. doi: 10.1371/journal.pcbi.1005634 (PMC5627952; doi:10.1371/journal.pcbi.1005634)

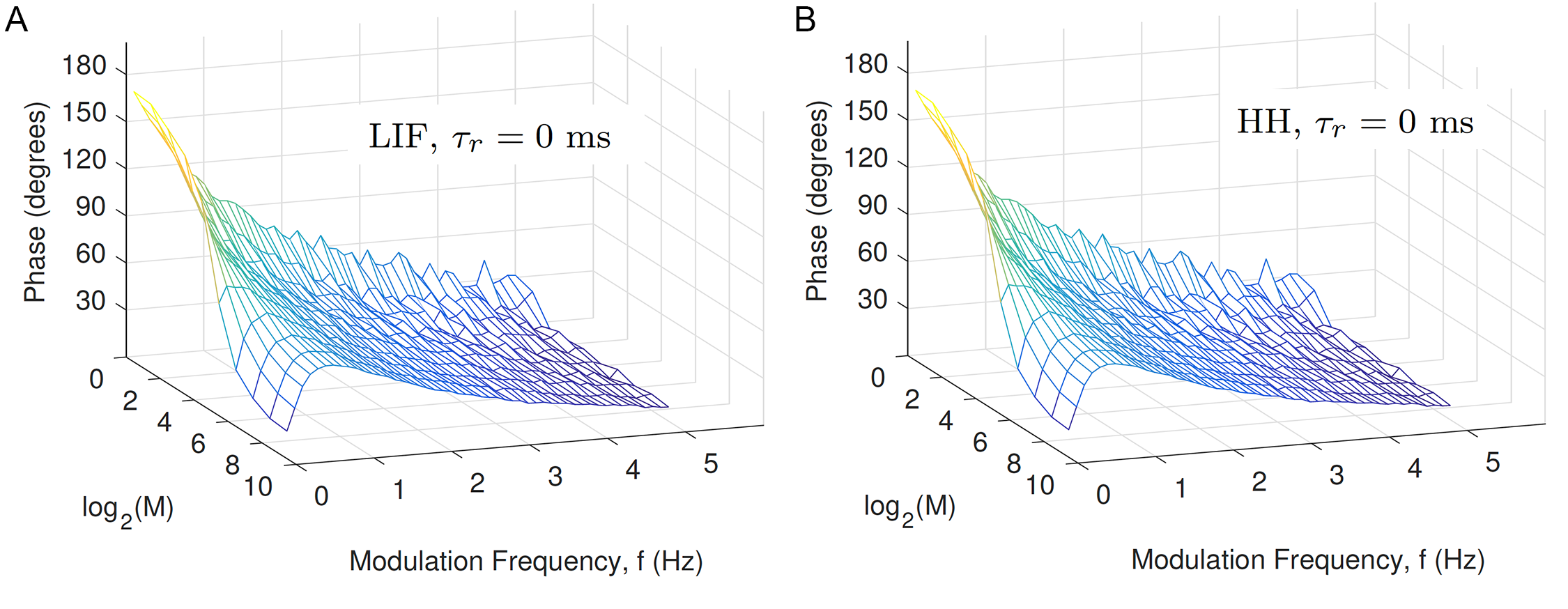

Supplement: S1 Fig — The trend as a function of modulation frequency and the number of pre-synaptic neurons, M, is very similar whether the output neuron is a leaky integrate-and-fire (LIF) model or Hodgkin-Huxley (HH). (TIFF) [file pcbi.1005634.s001.tiff]

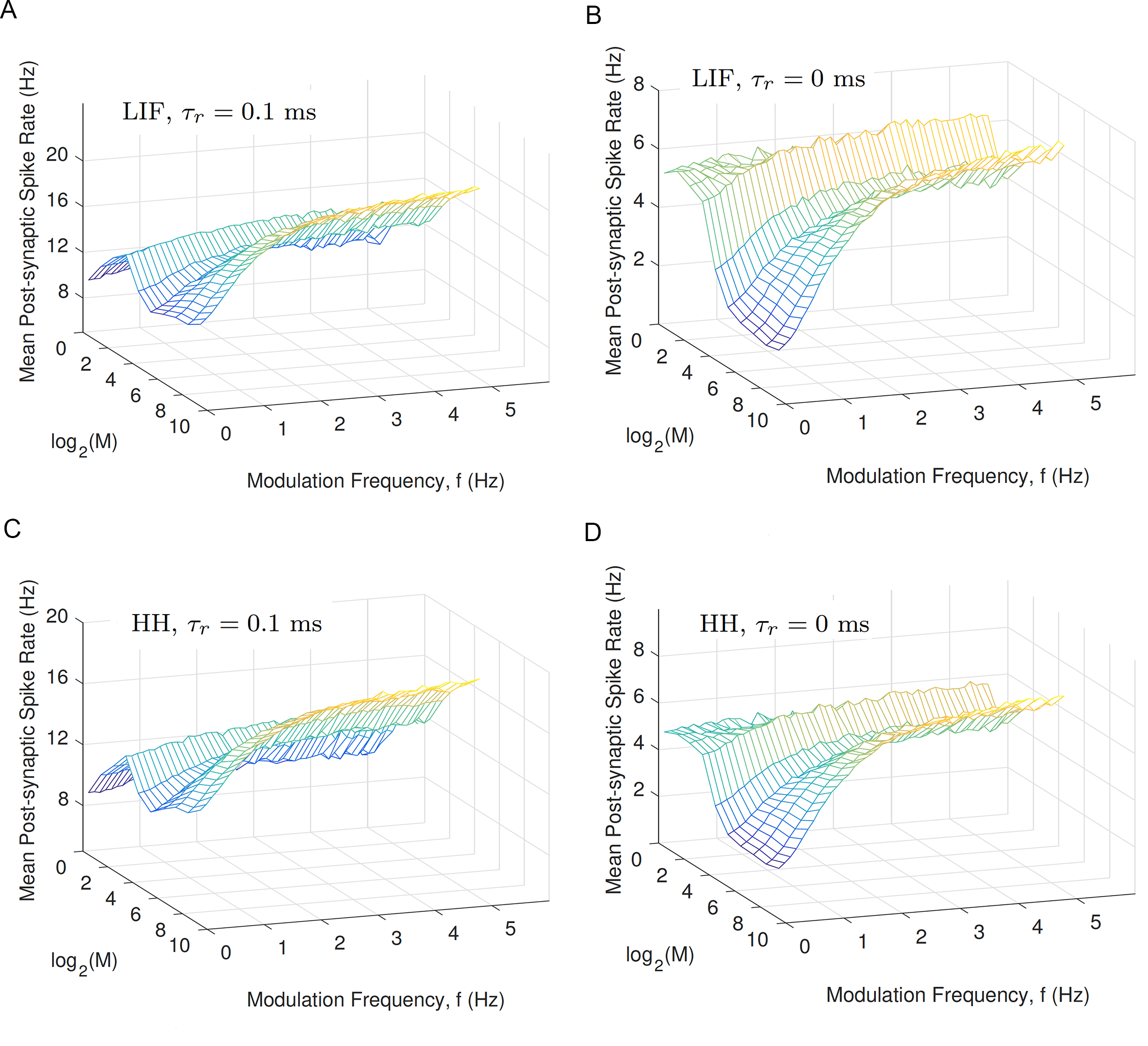

Supplement: S2 Fig — Unlike the phase lead, the spike rates observed and the manner in which they vary with modulation frequenecy and M, has a dependence on the model and whether the post-synaptic conductance change has a zero or non-zero rise-time. (TIFF) [file pcbi.1005634.s002.tiff]

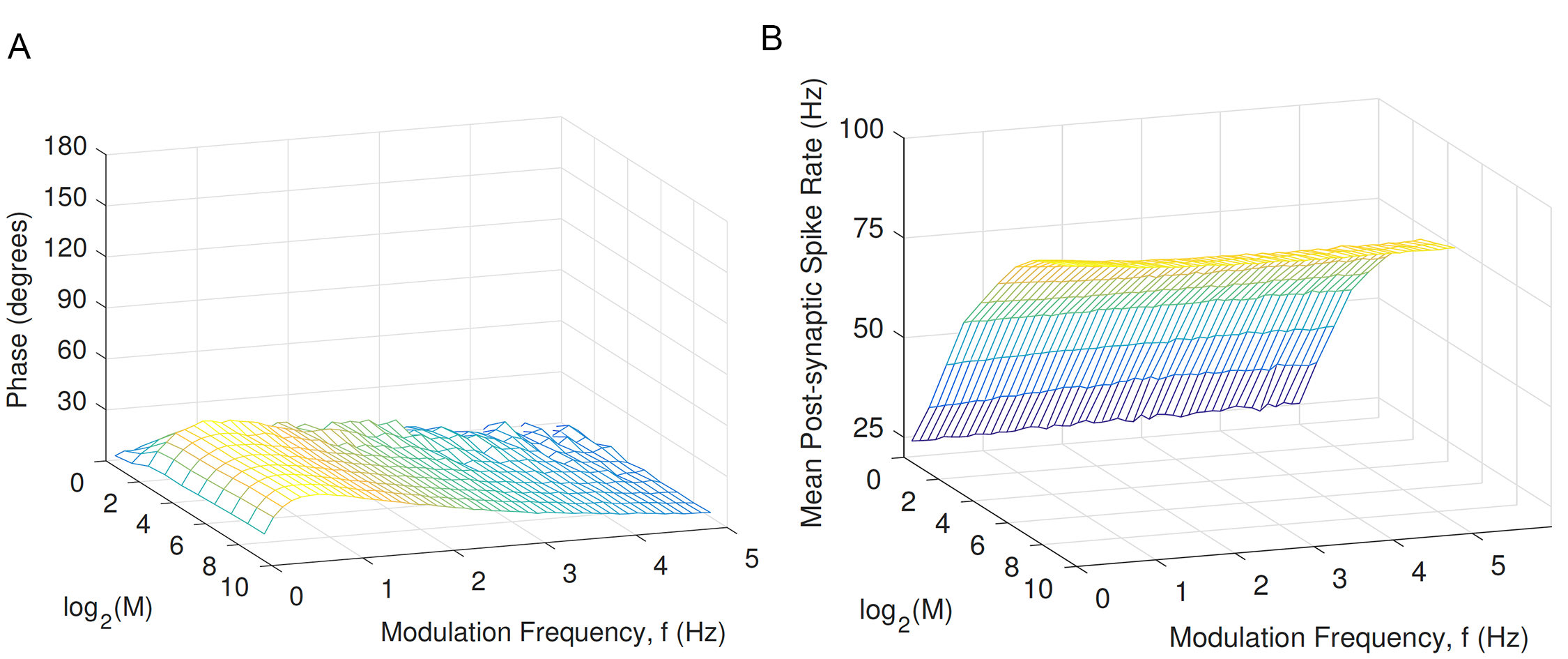

Supplement: S3 Fig — The model used is otherwise the same as the main text. The mean spike rate with such a long time constant becomes very large relative to τd = 1 ms, and the general trend of the phase now remains similar for all M due to increased temporal integration, even for low M. (TIFF) [file pcbi.1005634.s003.tiff]
